# Relationship Between National Economic Development and Body Mass Index in Chinese Children and Adolescents Aged 5–19 From 1986 to 2019

**DOI:** 10.3389/fped.2021.671504

**Published:** 2021-04-27

**Authors:** Te Bu, Stevo Popovic, Huiqing Huang, Tao Fu, Jovan Gardasevic

**Affiliations:** ^1^Faculty of Sport and Physical Education, Hunan Normal University, Changsha, China; ^2^Faculty for Sport and Physical Education, University of Montenegro, Niksic, Montenegro; ^3^Montenegrin Sports Academy, Podgorica, Montenegro; ^4^Faculty of Health and Exercise Science, Tianjin University of Sport, Tianjin, China

**Keywords:** obesity, overweight, gross domestic product, economic growth, supply-side economy

## Abstract

Obesity represents a major risk factor for population health. No studies have evaluated how economic expansion impacts the prevalence of obesity. The purpose of this study was to assess the relationship between national economic development and body mass index (BMI) in Chinese children and adolescents. Data of mean BMI in children and adolescents aged 5–19 from 1986 to 2019 were extracted from an international database of cardiometabolic risk factors. Chinese economic development was quantified by the gross domestic product (GDP), which was extracted from the International Monetary Fund. The relationships between GDP and BMI were assessed in 1-year age groups for ages 5–19 years. In addition, the linear regression from the main data and estimated GDP growth allowed the projections of mean BMI for each age group between 2020 and 2025. The results suggest there was a linear increase in BMI over years, which means that there has been a steady increase in BMI over the economic expansion. Overall, 97% of the variance (Pearson correlation coefficient) of BMI in boys can be explained by the GDP expansion, and the same pattern (98% of the variance) occurred in girls. Projected mean BMI were provided for constructing future national strategies to prevent overweight and obesity in youth. In conclusion, BMI in children and adolescents aged 5–19 trended upwards between 1986 and 2019. Our analyses for the first time suggest that globalization has a major impact on BMI in China. Economic expansion was highly predictive of BMI increases.

## Introduction

Obesity has emerged a global health problem and posed health risks to the world's population of all ages. According to the World Health Organization, the worldwide prevalence of obesity has tripled between 1975 and 2020 (1). In 2016, more than 1.9 billion adults aged 18 years and over were overweight, whereas over 340 million children and adolescents aged 5–19 were overweight or obese (1). More recently, data have shown that 38 million children worldwide under the age of 5 were overweight or obese in 2019 (1). The prevalence of overweight and obesity in childhood is high and continues to increase globally (2–5), generating an increasing health risk. The same trend is occurring in China, which is facing this major public health concern. Between 1995 and 2014, Chinese children and adolescents aged 7–18 saw a four-fold increase in overweight and obesity, with around 1 in 5 children and adolescents being either overweight or obese (6). Population data show that prevalence of overweight continually increased from 1.1% in 1985 to 20.4% in 2014 in Chinese school-aged children (7), making childhood obesity a critical health issue in China.

The etiology of obesity is complex. Although the genetic susceptibility to obesity is high (8), the rising global prevalence of obesity highlights behavioral changes associated with modern lifestyles (e.g., excessive intake of simple carbohydrates and sugar, physical inactivity) that contribute to its development (9, 10). A vital effect that has not yet been reported in the literature is the role of economic expansion in the development of overweight and obesity. For example, it has been suggested that rising obesity can be attributed to factors related to globalization processes, which can be related to flooding developing economies with western-style, inexpensive but obesogenic fast foods (11).

However, current literature shows conflicting results about the relationship between economic development and obesity. Rapid emergences of overweight and obesity are widely documented, even in the poorest countries of sub-Saharan Africa (12), which exhibit little economic expansion in the past decade. In general, Seydel et al. (13) suggested that economic growth leads to increase of overweight and obesity in developing countries. Specifically, 1% increase in income leads to around a 0.2 and 0.3% increase in the overweight and obesity prevalence, respectively (14). For instance, compared with adult women living in poorest households in Nepal, the odds of being overweight were, respectively 3.44, 2.12, 1.46, and 1.19 times higher for women living in richest, richer, middle and poorer wealth status households (15). As a result, accompanying with rapid economic growth, emerging economies are undergoing a noticeable shift in the disease structure marked by increasingly higher proportion of non-communicable disease-related morbidities (6, 16). However, among developed economies, higher gross domestic product (GDP) predicted lower body mass index (BMI) (11).

Coincident with economic development, China has experienced a marked transition from undernutrition to overweight and obesity since the reform and opening up of Chinese economy (6, 7). An important step in addressing the growing overweight and obesity trend in China, as well as in the global community where obesity has literally become an epidemic, is understanding all key factors, including national economic development, associated with childhood and adolescent obesity. Given the critical impact that childhood and adolescent obesity has on lifetime health (17), it is crucial to explore the relationship between rapid expansion of the Chinese economy and BMI, the most commonly used measure for assessing obesity. Therefore, the purpose of this study was to evaluate the relationship between an economic factor (GDP) and BMI in children and adolescents aged 5–19 in China. In addition, we projected the trend change in BMI between 2020 and 2025, which should offer national policy makers to design more targeted strategies to combat youth overweight and obesity. This study is first of its kind in China.

## Methods

### Data Sources

BMI data were available from an international database on cardiometabolic risk factors managed by the Non-Communicable Disease Risk Factor Collaboration (18). This database is continuously updated and national data (i.e., mean BMI of China Mainland) used in this study were extracted as of 31 December 2020. While the total sample sizes for country specific data are not available, BMI data collected by the Non-Communicable Disease Risk Factor Collaboration currently consist of 2,181 population-based studies with 65 million participants, which is considered to be the most comprehensive international scientific endeavor documenting trend change in BMI. GDP data of China Mainland between 1986 and 2019 were available from the International Monetary Fund (19).

### Statistical Analysis

Since linear function yielded same results as the first order polynomial function, we used linear regression to explore the relationship between GDP and BMI. Pearson correlation coefficients were used to quantify associations between GDP and BMI. Linear regression for each age group allowed us to project the trend development of BMI. We used a real GDP annul growth of 2.3% for 2020 (19), and projected real GDP annul growth of 6.0% between 2021 and 2025 (20) to calculate the GDP growth and subsequently projected change in BMI from 2020 to 2025. Prism 9 (GraphPad Software, San Diego) was used to conduct the analyses in this study.

## Results

**Figure 1:**
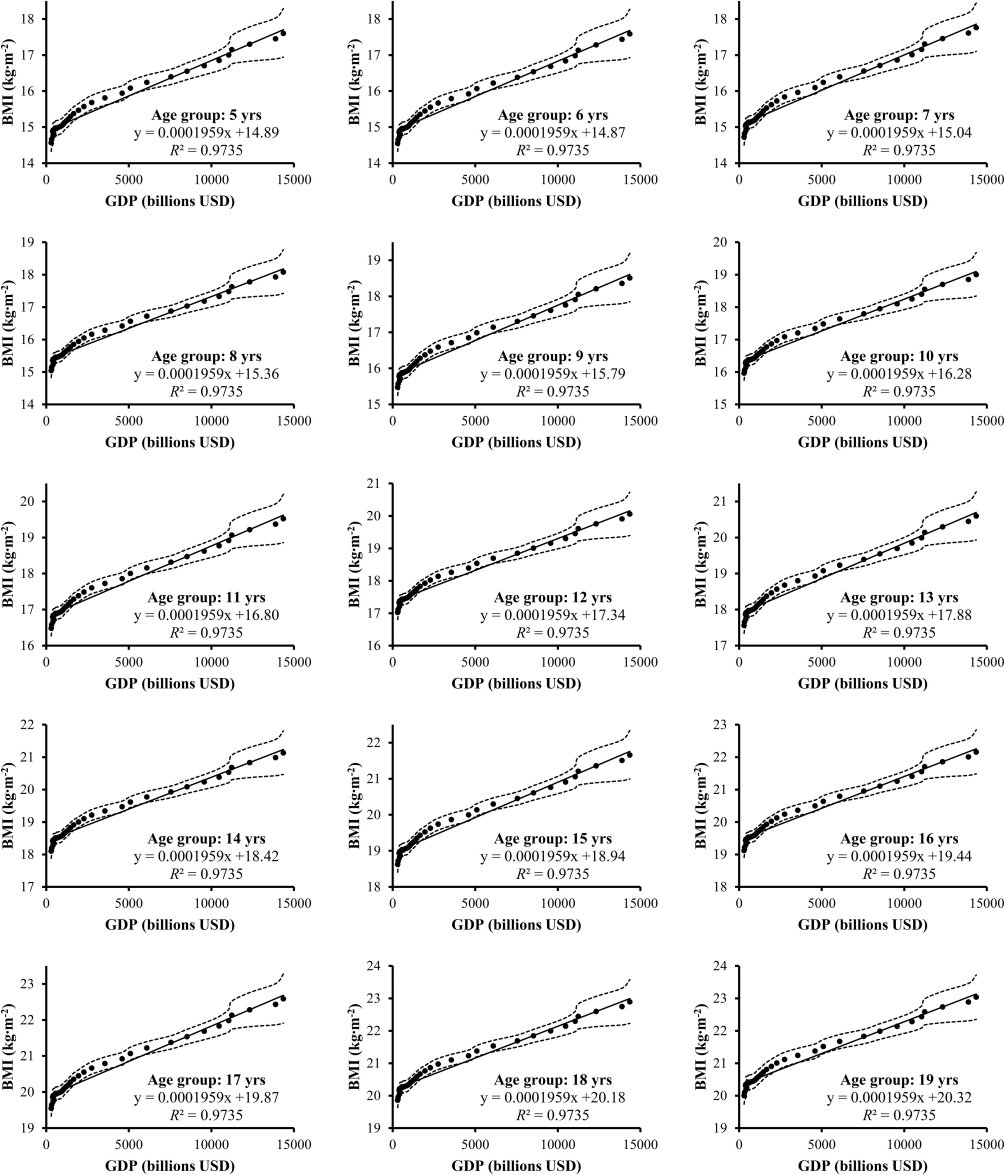
Scatter plots with linear regression lines of gross domestic product (GDP) and body mass index (BMI) of Chinese boys aged 5–19 with respective Pearson correlation coefficients. Dotted lines represent 95% confidence interval.

**Figure 2:**
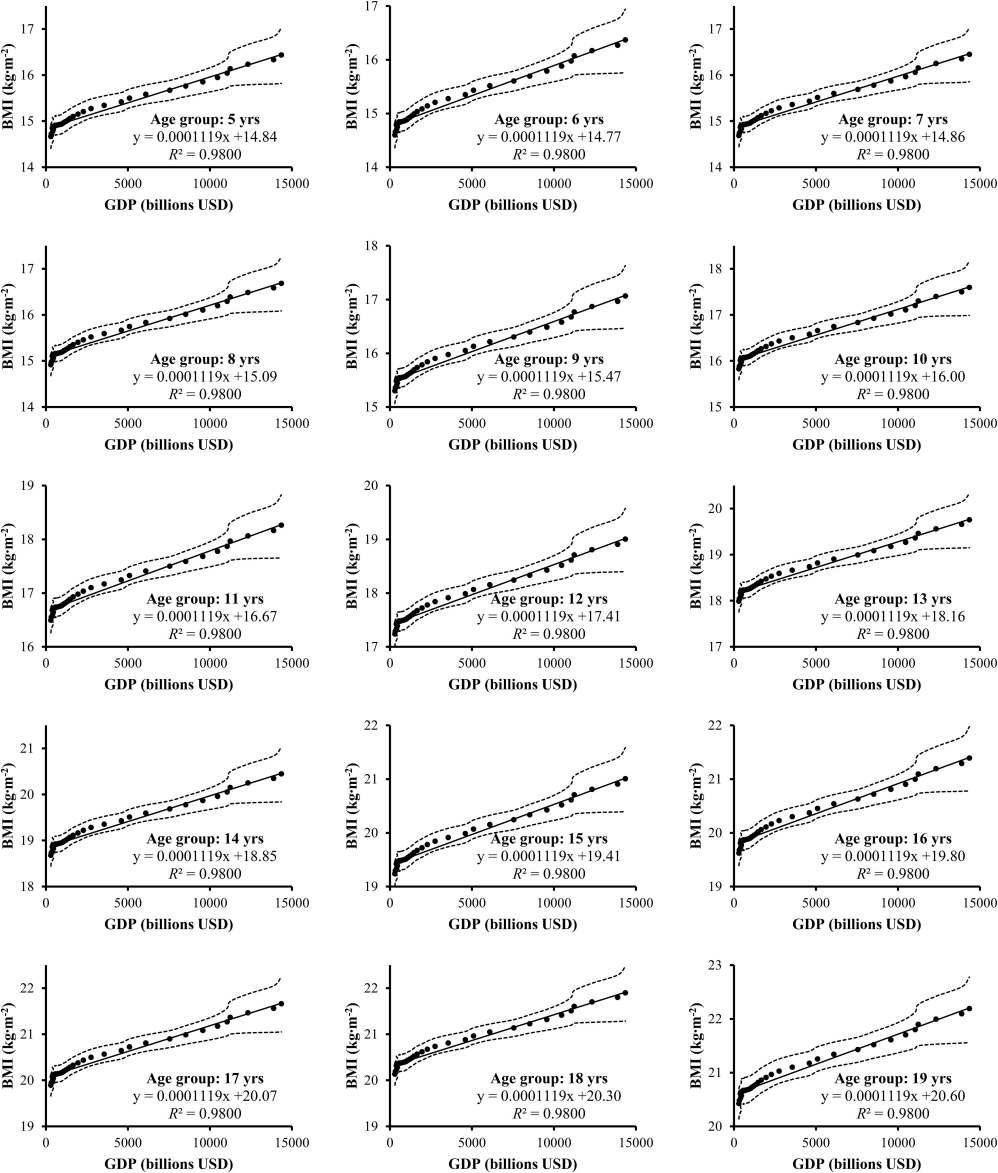
Scatter plots with linear regression lines of gross domestic product (GDP) and body mass index (BMI) of Chinese girls aged 5–19 with respective Pearson correlation coefficients. Dotted lines represent 95% confidence interval.

Scatter plots with respective correlation coefficients and linear regression lines are depicted in Figures 1, 2. There has been a steady increase in BMI over the measured 35 years. Linear regression revealed that GDP expansion was associated with increased BMI in children and adolescents over the time. In general, this pattern was seen in both boys (*R*^2^ = 97%) and girls (*R*^2^ = 98%).

Using the economic growth model, projected BMI in 2020–2025 is shown in Table 1. Overall, it is projected that economic expansion over the next 6 years could yield around 0.80 and 0.51% annual increase in BMI occurrence for boys and girls aged 5–19, respectively.

**Table 1:** Projected mean body mass index of Chinese children and adolescents aged 5–19 between 2020 and 2025.

**Age group (years)**	**2020 BMI** **(kg·m**^****−2****^**)**		**2021 BMI** **(kg·m**^****−2****^**)**		**2022 BMI** **(kg·m**^****−2****^**)**		**2023 BMI** **(kg·m**^****−2****^**)**		**2024 BMI** **(kg·m**^****−2****^**)**		**2025 BMI** **(kg·m**^****−2****^**)**	
	**Boys**	**Girls**	**Boys**	**Girls**	**Boys**	**Girls**	**Boys**	**Girls**	**Boys**	**Girls**	**Boys**	**Girls**
5	17.8	16.5	17.9	16.6	18.1	16.7	18.3	16.8	18.5	16.9	18.7	17.0
6	17.7	16.4	17.9	16.5	18.1	16.6	18.3	16.7	18.5	16.8	18.7	17.0
7	17.9	16.5	18.1	16.6	18.3	16.7	18.5	16.8	18.7	16.9	18.9	17.1
8	18.2	16.7	18.4	16.8	18.6	16.9	18.8	17.0	19.0	17.2	19.2	17.3
9	18.7	17.1	18.8	17.2	19.0	17.3	19.2	17.4	19.4	17.5	19.6	17.7
10	19.2	17.6	19.3	17.7	19.5	17.8	19.7	18.0	19.9	18.1	20.1	18.2
11	19.7	18.3	19.9	18.4	20.0	18.5	20.2	18.6	20.4	18.7	20.7	18.9
12	20.2	19.1	20.4	19.2	20.6	19.3	20.8	19.4	21.0	19.5	21.2	19.6
13	20.8	19.8	20.9	19.9	21.1	20.0	21.3	20.1	21.5	20.2	21.7	20.4
14	21.3	20.5	21.5	20.6	21.7	20.7	21.8	20.8	22.1	20.9	22.3	21.0
15	21.8	21.1	22.0	21.2	22.2	21.3	22.4	21.4	22.6	21.5	22.8	21.6
16	22.3	21.4	22.5	21.5	22.7	21.6	22.9	21.8	23.1	21.9	23.3	22.0
17	22.7	21.7	22.9	21.8	23.1	21.9	23.3	22.0	23.5	22.1	23.7	22.3
18	23.1	21.9	23.2	22.0	23.4	22.1	23.6	22.3	23.8	22.4	24.0	22.5
19	23.2	22.2	23.4	22.3	23.6	22.4	23.7	22.6	24.0	22.7	24.2	22.8

*BMI, body mass index*.

## Discussion

This is the first study of the relationship between national economic development and BMI in China. Results from this study show for the first time that higher BMI in both boys and girls and in all age groups between 5 and 19 was strongly related to the economic expansion in the past 35 years in China. From the epidemiological point of view, the increase in BMI was related to the rapid and strong economic growth in China, which should constitute an important public health concern in modern China.

The current study aligns with substantial evidence that overweight and obesity among Chinese school-aged children and adolescents are trending upwards since the twenty-first century. For instance, Zhang et al. (21) found that between 2010 and 2015, the age-adjusted prevalence of overweight and obesity among boys increased from 21.2 to 31.7% and from 10.6 to 16.9% among girls. As the evidence accrues, this study first provides a readily available macro-indicator for explaining temporal trends of overweight and obesity in China. Very importantly, this study allows a projection of BMI. We estimate by 2025, BMI will increase to 21.1 kg·m^−2^ for boys and 19.6 kg·m^−2^ for girls in China. This can allow public health administrators to construct more targeted policy interventions for the prevention of overweight and obesity among youth. Specifically, relevant governing bodies in China can establish expert panels consisting of policy makers and health professionals and researchers, to construct national strategies on combating negative consequences related to the trending childhood overweight and obesity in China. Should this upwards trend of BMI among the young population be controlled, not only it could be translated to reduced health burdens for the society as a whole, but it could also further boost the Chinese economy in the long run as suggested in an economic model by Kelly et al. (22). Hence, the current results could be very important.

Although the overall BMI is still in the normal range, the physiological impact of overweight and obesity is greater among Chinese population compared to westerners. At the same BMI, risks for the development of metabolic and cardiovascular diseases are elevated for Chinese ethnicities compared to westerners (23, 24). The risk for developing hypertension is doubled in Chinese with BMI between 23.0 and 24.9 kg·m^−2^ and tripled with BMI between 25.0 and 26.9 kg·m^−2^ (25). Therefore, BMI cut points for overweight and obesity in Asians were lowered to 23.0–27.4 kg·m^−2^ and ≥27.5 kg·m^−2^, respectively, compared to standard BMI cut points for overweight (25.0–29.9 kg·m^−2^) and obese (≥30.0 kg·m^−2^) in western nations (26).

Based on the trend analyses, BMI among the young Chinese population continues to increase, and this trend may be more important than ever for health providers and national policy makers. It is expected that the Chinese economy will continue to expand at 6% annual rate (20), and according to this study's regression model, this economic growth rate will be translated to 0.80 and 0.51% annual increase in BMI for Chinese boys and girls, respectively. Thus, healthy weight management and obesity prevention protocols should be included in the national education system and those establishing the BMI cut-off point used to identify obesity need to carefully consider the Chinese specific difference in relation to the cardiovascular risk spanning through childhood and adulthood.

It should be notated that a correlation does not allow for causal inference but can provide a framework to guide future investigations and interventions. In our study, the Chinese GDP expansion was highly predictive of BMI increase in Chinese children and adolescents, which is in line with findings from other developing economies (15, 16). However, overweight and obesity are also growing in countries where GDP are not growing continuously (12). Furthermore, as countries develop economically, overweight prevalence remains mostly unchanged among the wealthiest countries (27). This should not be surprising though. Increase in population BMI is driven by many factors, including the human development index (HDI) (14). HDI is a summary measure of average achievement in key dimensions of human development, including a long and healthy life, being knowledgeable and have a decent standard of living (28). Although rising income per capita tends to push populations toward unhealthy western eating habits, in the very high HDI countries, there is a positive effect on eating habits of any further improvement in per capita income (14). China have witnessed a marked transition in its diet, activity and nutritional status patterns during the rapid economic expansion. According to the latest “Report on Nutrition and Chronic Disease Status of Chinese Residents (2020)” (29), the Chinese dietary pattern has shifted toward a dietary pattern with high consumption of meats and edible oil but low consumption of cereals and vegetables. Children and teenagers' frequent drinking of sugary beverages and insufficient physical activity are widespread. The net effect of all these changes in diet has been to increase in total energy intake and consequently higher prevalence in overweight and obesity among Chinese residents. Taken collectively, there is a pressing need for national policy adjustments to extend beyond an emphasis on rapid economic growth alone, and to campaign on healthy diets and lifestyles to achieve synchronized improvement in HDI.

As has been mentioned in the methods, the present study has an inherent limitation from data source. The study used BMI data from an international database where neither the sample sizes nor the quality of these data collection can be verified independently. It is possible that not all studies were of similar quality in terms of randomization and convenience sampling. Thus, the impact of these limiting factors on the current analysis is unclear. However, the Non-Communicable Disease Risk Factor Collaboration is internationally recognized databases that collect data from peer-reviewed literatures. Nonetheless, we acknowledge the present conclusion may change as future studies appear in the literature and merit future continuous investigation.

Although the causation for the relationship between GDP and BMI is beyond the scope of this study, our data provide the first insight on how globalization impacts BMI. An important implication is that resolving this health epidemic should not only rely on traditional health-based guidelines. The present results could help launch a comprehensive national obesity initiative, while laying the foundations for physical well-being into 2021–2025. Future task forces for designing pediatric practice should also attempt to explore the influence of targeted economic activities.

From the macro-perspective, the present results could offer novel insights to reverse-engineer a positive economic impact that stimulates not only supply-side economy but also demand-side related social changes. We recommend health professionals and economists jointly assess how reduced reliance on ultra-processed foods related to globalization could yield positive economic and health outcomes. For instance, the recent booming of fresh food e-commerce in China, a market valued at ~200 billion Chinese Yuan in 2020 (30), may provide an excellent opportunity to better control the curve of population weight gain, should consumer behaviors be driven to the consumption of healthy fresh foods. Together with traditional promotion of active lifestyles (e.g., daily walking for 8,000 steps per day), comprehensive nationwide interventions containing novel economic approaches could creatively reduce overweight and obesity whilst stimulating the high-quality, health-related consumer economy in China.

## Conclusion

China was, and still is, one of the fastest growing economies in the world. With obesity on the rise across the nation, plus an increased risk at lower BMI cut points among Chinese ethnicities for chronic diseases, it is important to acknowledge and understand how rapid economic expansion affected this process. This study's findings on the strong linear relationship between national economic development and BMI highlight the need for alternative economic interventions and strategies tailored for the young Chinese population to control overweight and obesity through adulthood. We expect that a carefully planned, innovative approach could simultaneously help change consumer behaviors and yield enhanced health outcomes and economic growth in modern China.

## Data Availability Statement

The raw data supporting the conclusions of this article will be made available by the authors, without undue reservation.

## Author Contributions

TB and SP contributed conception and design of the study. HH and TF helped design and coordinate data collection. TB, HH, and TF performed the statistical analyses and wrote the first draft of the manuscript. SP and JG critically reviewed and edited the manuscript. All authors contributed to manuscript revision, read, and approved the submitted version.

## Conflict of Interest

The authors declare that the research was conducted in the absence of any commercial or financial relationships that could be construed as a potential conflict of interest.
